# In Vivo Confocal Microscopy Revealed Alteration of Limbal Stem Cells and Sub-Basal Nerve Plexus in Allergic and Dry Eye-Related Ocular Surface Diseases

**DOI:** 10.1167/tvst.15.7.7

**Published:** 2026-07-08

**Authors:** Xiaojie Wan, Kaiye Zhang, Yu Zhang, Yujie Mou, Xiaodan Huang

**Affiliations:** 1 Eye Center, The Second Affiliated Hospital, School of Medicine, Zhejiang University, China; 2 Zhejiang Provincial Key Laboratory of Ophthalmology, Zhejiang Provincial Clinical Research Center for Eye Diseases, Zhejiang Provincial Engineering Institute on Eye Diseases, Hangzhou, China; 3 Department of Ophthalmology, Peking University Third Hospital, Beijing Key Laboratory of Restoration of Damaged Ocular Nerve, Peking University Third hospital, Beijing, China

**Keywords:** palisades of Vogt, sub-basal nerve plexus, vernal keratoconjunctivitis, blepharokeratoconjunctivitis, allergic conjunctivitis, IVCM

## Abstract

**Purpose:**

To evaluate alterations of the palisades of Vogt (POV), basal epithelial cell density (BECD), and the corneal sub-basal nerve plexus (SBNP) in patients with vernal keratoconjunctivitis (VKC), blepharokeratoconjunctivitis (BKC), and allergic conjunctivitis (AC) using in vivo confocal microscopy (IVCM).

**Methods:**

IVCM imaging of the limbus, central basal epithelial layer, and SBNP was performed in 122 participants (39 VKC, 26 BKC, 42 AC, and 14 normal controls). POV was graded semiquantitatively. To account for intereye correlation, generalized estimating equations were applied, and ordinal logistic regression was used to identify factors associated with POV visibility. Spearman correlation analysis was performed to evaluate relationships between SBNP parameters, BECD, and POV scores.

**Results:**

POV visibility was significantly associated with age, disease group, disease duration, and limbal quadrant. Compared with controls, patients with VKC, BKC, and AC showed significantly lower POV scores, accompanied by decreased BECD and SBNP parameters. Older age and longer disease duration were associated with lower POV scores; a more pronounced POV morphology was observed in the superior and inferior quadrants. Significant correlations were observed among BECD, SBNP parameters, and POV scores.

**Conclusions:**

Chronic ocular surface inflammation may be associated with disruption of the limbal stem cell niche, reflected by alterations in POV, cellular, and neural parameters, supporting the potential role of IVCM-derived metrics in the early detection of limbal stem cell dysfunction.

**Translational Relevance:**

Vivo confocal microscopy may provide noninvasive in vivo biomarkers of limbal niche and corneal nerve alterations in inflammatory ocular surface diseases, with potential value for earlier identification of limbal stem cell deficiency–related changes.

## Introduction

Inflammation-mediated limbal damage^1^ is a recognized contributor to limbal stem cell deficiency (LSCD).^2^ Chronic ocular surface inflammatory diseases, particularly allergic and blepharitis-related conditions, may lead to progressive disruption of the LSC niche and subsequent corneal epithelial abnormalities. Vernal keratoconjunctivitis (VKC)^3^ is a severe and recurrent form of allergic conjunctivitis (AC) that predominantly affects preadolescent children and is characterized by chronic inflammation with seasonal exacerbations. Blepharokeratoconjunctivitis (BKC)^4^ is a chronic inflammatory disorder of the eyelid margin associated with secondary corneal involvement, including superficial punctate erosions, corneal infiltration, ulceration, and corneal vascularization.^5^ In comparison with adult patients, children with BKC typically suffer from poorer visual outcomes and more severe corneal disorders, potentially due to delayed diagnosis.^6^ In addition, AC,^7^ including seasonal and perennial subtypes, represents a common ocular surface inflammatory condition that can significantly affect patients’ quality of life. Despite the shared inflammatory basis of these conditions, their effects on corneal microstructure, particularly at the level of the LSC niche, remain incompletely understood.

The LSC niche refers to a specialized microenvironment composed of epithelial, stromal, and neural components, along with extracellular matrix and regulatory factors, that collectively maintain stem cell function and corneal epithelial homeostasis.^8^ The palisades of Vogt (POV), characterized by limbal epithelial crypts and focal stromal projections, are widely regarded as an important component of the LSC niche.^9^ Damage to LSCs within the POV may result in persistent epithelial damage and corneal conjunctivization, resulting in turbidity, inflammation, neovascularization, and chronic scarring.^10^

In addition to structural alterations of the POV, changes in basal epithelial cell density (BECD) and the sub-basal nerve plexus (SBNP) have been considered as important in vivo indicators of LSCD.^11^ An average of 23.5% to 56.2% reduction of BECD has been reported in both the central cornea and limbus in patients with LSCD, indicating early impairment of LSC function.^12^ Corneal nerves are essential for maintaining epithelial integrity and wound healing,^13^ and alterations in the SBNP, which is situated between the basal epithelial cells and the Bowman layer, have been observed in various ocular and systemic conditions, including acute *Acanthamoeba* and fungal keratitis,^14^ dry eye disease,^15^ congenital corneal anesthesia,^16^ keratoconus,^17^ ametropia, orthokeratology lens wearers,^18^ diabetes,^19^ and Parkinson's disease.^20^ The SBNP can be considered as an indicator of corneal health and/or the status of the peripheral nervous system status. Meanwhile, the SBNP can be related to the severity of LSCD according to the research of Le et al.,^11^ as sensory nerve depletion reduces LSCs and compromises the stem cell niche.^21^

In vivo confocal microscopy (IVCM) has become an important tool in the evaluation of corneal diseases.^22^ This noninvasive technique enables high-resolution visualization of corneal cellular architecture and has been widely used to assess POV, BECD, and SBNP. In this study, we used IVCM to investigate alterations in POV, BECD, and SBNP in patients with VKC, BKC, and AC. We hypothesized that chronic inflammatory ocular surface diseases are associated with structural changes in the LSC niche, reflected by reduced POV visibility and concurrent alterations in epithelial and neural parameters, and that these changes may vary according to disease characteristics, such as duration and limbal location.

## Methods

The recruitment process was conducted at the outpatient clinics of the Second Affiliated Hospital, Zhejiang University School of Medicine in 2021 and 2022, in accordance with Clinical Practice guidelines and the tenets of the Declaration of Helsinki. The study was approved by the institutional review board of the Second Affiliated Hospital, Zhejiang University School of Medicine, Hangzhou, China (identifier: 20210403). The study included 122 participants, comprising 39 patients with VKC, 26 with BKC, 42 with AC, and 14 normal controls, and they were enrolled according to the following eligibility criteria. All participants signed an informed consent form.

### Patient Identification and Classification

Normal controls were individuals with no history of ocular disease, no ocular surface symptoms, and normal findings on slit-lamp biomicroscope and ocular surface examinations.

The diagnostic criteria for VKC^3^ included the presence of a foreign body sensation and tearing in both eyes, accompanied by itching. Ovate papillae hyperplasia could be observed under slit-lamp examination in the conjunctiva of the upper eyelid, resulting in a classic cobblestone appearance. Alternatively, gelatinous, confluent, yellow–gray infiltrates known as Horner–Trantas dots could be observed in the cornea limbus. Disease severity was further categorized based on characteristic clinical signs reported in previous studies,^23^ including active giant papillae, gelatinous infiltrates of the limbus, exfoliative epithelial keratopathy, shield ulcer, and papillary proliferation at the lower palpebral conjunctiva. Patients presenting with one or none of these signs were classified as having mild-to-moderate disease, whereas those with two or more signs were classified as having severe disease.

The diagnostic criteria for BKC^4^ included symptoms of recurrent chalazia, ocular irritation, tearing, chronic discomfort, photophobia, foreign body sensation, blurred vision, and red eye. Typical signs in the lid margin included meibomitis, meibomian gland dysfunction, eyelid inflammation, lid erythema, chalazion, and hordeola; in the conjunctiva, conjunctival hyperemia, conjunctivitis, and phlyctenules; and in the cornea, corneal infiltrates, scarring, vascularization, thinning, ulcer, pannus, phlyctenules, and superficial punctate keratitis. The diagnosis of BKC requires one or more suggestive symptoms (or history) plus one or more clinical signs from each of the three anatomical regions. Patients diagnosed with both blepharitis and keratoconjunctivitis, but with ocular surface involvement that was not the consequence of blepharitis, were carefully excluded. Disease severity in BKC was classified based on the extent of corneal involvement, according to previously reported clinical criteria.^24^ Patients were categorized as having mild-to-moderate disease when only mild corneal involvement was present, such as punctate keratopathy or marginal infiltration, whereas severe disease was defined by more advanced corneal involvement, including ulceration, vascularization, or scarring.

A diagnosis of AC^7^ was established based on clinical history and characteristic signs and symptoms, including conjunctival hyperemia, chemosis, ocular itching, and increased ocular discharge. Allergen sensitization was assessed using a standard serum-specific immunoglobulin E test panel^25^ (including common environmental allergens such as dust mites, pollen, and animal dander), which served as a supportive diagnostic tool. The AC group included patients with seasonal AC and perennial AC, and VKC was analyzed as a separate entity. Although seasonal AC and perennial AC may differ in disease course and inflammatory characteristics, they were analyzed as a single AC group, because the primary aim of this study was to compare VKC with non-VKC allergic conditions. For patients with AC, disease severity was categorized based on characteristic clinical signs reported in previous studies,^23^ including blepharitis, papillary proliferation with a velvety appearance, Horner–Trantas spots, edema of the bulbar conjunctiva, and superficial punctate keratopathy. Patients presenting with two or fewer of these signs were classified as having mild-to-moderate disease, whereas those with three or more signs were classified as having severe disease.

### Ophthalmic Examination

A medical history and demographic information of patients with AC, VKC, BKC, and the normal controls were collected. Routine ophthalmic examinations were performed, including slit-lamp biomicroscope, anterior segment photography (corneal fluorescein sodium staining), tear meniscus height, tear film breakup time, and dry eye infrared examination using OCULUS keratography (OCULUS, Wetzlar, Germany).

### Confocal Imaging

IVCM (HRT-III, Heidelberg Engineering, Heidelberg, Germany)^26^ was used to image the morphological characteristics of the cornea. The device uses confocal scanning laser microscopy to provide high-resolution images of the cornea and other external eye structures, such as the conjunctiva or limbus. By scanning the cornea with a field of vision of up to 400 × 400 µm, the device allowed for the acquisition of detailed frontal images of the corneal cells and structures. This process enables the identification of different corneal cellular layers and cell types, including epithelial wing cells, basal epithelial cells, and stromal keratocytes, as well as visualization of the SBNP, as previously described.^26^ The entire procedure was performed by an experienced technician with at least 3 years of experience in IVCM imaging. Before the examination, a sterile disposable corneal contact cap (TomoCap) was mounted on the objective lens to maintain optical coupling and prevent contamination, which was then applied with a drop of carbomer gel (Bausch & Lomb, Berlin, Germany). Subsequently, a drop of topical anesthetic (0.5% proparacaine hydrochloride Eye Drops; Alcon, Fort Worth, TX) was administered to the eye. Participants were instructed to fixate on predefined targets to systematically guide gaze, allowing imaging of the limbal region in superior, inferior, nasal, and temporal quadrants. A minimum of five clear, high-quality, nonoverlapping, and representative photographs were captured in the four limbal quadrants.

### Tacrolimus Eye Drops Treatment

Patients diagnosed with VKC were treated with tacrolimus eye drops (one drop twice a day per eye). Follow-up examinations were performed at baseline and at 4 months after treatment, and the assessment protocol was identical to that used at baseline, including routine ophthalmic examinations and IVCM.

### Image Analysis

**Figure 1.**
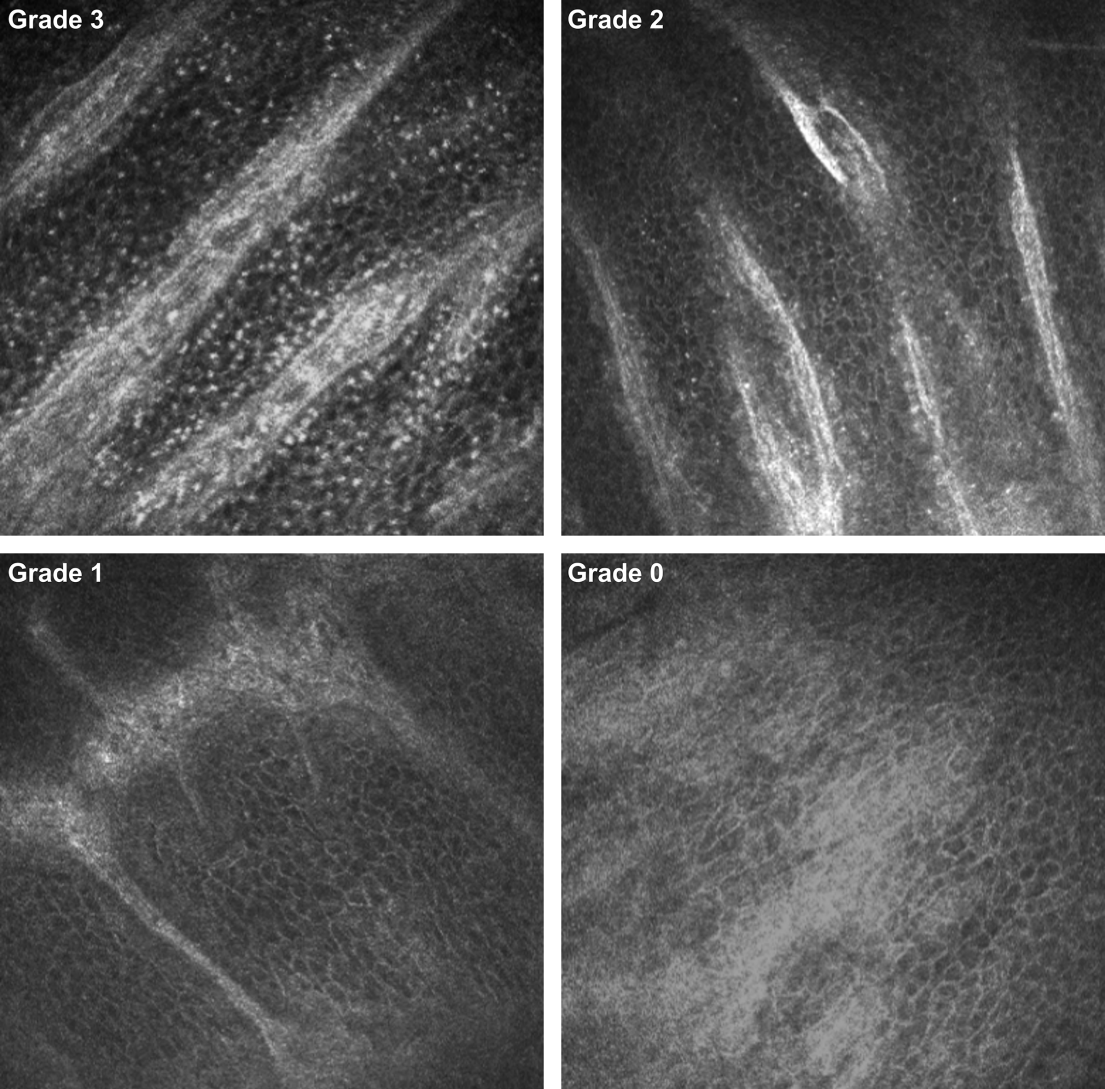
POV grading scale under IVCM. (Grade 3) Marked prominence, with wavy epithelial–stromal borders or alternating epithelial–stromal cords with bright, highly reflective basal epithelial cell margins. (Grade 2) Moderate prominence, with alternate epithelial–stromal cords with reduced luminosity and blurred margins of highly reflective basal epithelial cells. (Grade 1) Slight prominence, with significantly reduced brightness and sparse placement of the highly reflective stromal cords, and no bright high-reflective basal epithelial cell margins. (Grade 0) No prominence, indicating neither alternate epithelial–stromal cords nor highly reflective stromal cords.

POV grading was performed by a trained and experienced observer who was masked to the clinical information of the participants. The semiquantitative grading of POV visibility (grades 0–3) was performed based on previously reported imaging-based grading approaches^27^ (Fig. 1). Grade 0 was classified by no prominence, indicating the absence of both epithelial–stromal reflective structures and highly reflective stroma cord-like structures. Grade 1 included a slight prominence, characterized by markedly reduced brightness and sparse distribution of stromal reflective cord-like structures, without clearly visible basal epithelial cell borders. Grade 2 featured a moderate prominence, with the presence of epithelial–stromal reflective structures showing reduced reflectivity and partially blurred basal epithelial cell borders. Grade 3 included a marked prominence, characterized by well-defined epithelial–stromal reflective structures with increased reflectivity and clearly delineated basal epithelial cell borders, often forming wavy epithelial–stromal interfaces.^28^ POV structures were graded in the superior, inferior, nasal, and temporal quadrants for each subject.

The morphology of the cells in the basal epithelial layers and of the nerves in the SBNP was examined. The basal epithelial cell layer was defined as the epithelial cell layer just above the SBNP. Three images showing clear morphology of the central basal epithelial cell layer were selected for BECD analysis. Similarly, five images that demonstrated the highest number of SBNPs were selected to assess the SBNP. SBNP parameters were quantified using ACCMetrics, an automated software for corneal nerve analysis.^29^ The parameters included nerve fiber density (NFD), nerve branch density (NBD), and nerve fiber length (NFL). The NFD refers to the number of main nerve fibers per unit area (fibers/mm²), the NBD is the number of branches arising from main fibers per unit area (branches/mm²), and the NFL represents the total length of all nerve fibers per unit area (mm/mm²).

### Statistical Analyses

Statistical analyses were performed using GraphPad Prism 9 (GraphPad Software, San Diego, CA) and IBM SPSS Statistics 27.0 (IBM Corp., Armonk, NY). Given that both eyes from some participants were included in the analysis, generalized estimating equations (GEEs) were applied to account for intereye correlations, with patient ID specified as the clustering variable. Ordinal logistic regression using GEEs was used to evaluate factors associated with the visibility score of the POV. The proportional odds assumption was assessed using a test of parallel lines and was not violated. In stratified analyses, lower POV scores were observed in participants with disease duration of 1 year or longer and in those with severe disease compared with the mild-to-moderate subgroup, with the greatest between-group difference identified in the superior limbal quadrant. For age-stratified analyses, participants were categorized into three groups based on their age at the time of examination: 10 years or less, 11 to 20 years, and more than 20 years.

The Kruskal–Wallis test^30^ was adopted to evaluate the differences in POV morphology and SBNP parameters between BKC, AC, and VKC patients and normal controls. If the *P* value was significant (*P* < 0.05), Dunn's multiple comparison tests^31^ were applied to assess the significance between the different groups. Spearman correlation analysis was used to analyze the correlation between SBNP parameters and BECD and POV scores. The Wilcoxon signed-rank test was applied to explore potential changes in limbal niche after tacrolimus therapy.

## Results

### Patients Characteristics

The demographic and clinical characteristics of both the normal control and disease groups are presented in Table 1. The study involved a total of 122 participants (209 eyes), including 39 VKC patients (70 eyes), 26 BKC patients (32 eyes), 42 AC patients (81 eyes), and 14 normal control (26 eyes). There were no significant differences in baseline characteristics (age and sex) among groups (age, *P* = 0.276; sex, *P* = 0.316).

**Table 1.** Demographic and Clinical Features of NCs and Disease Groups

Characteristic	VKC	BKC	AC	NC
No. of patients/eyes	40/70	26/32	42/81	14/26
Age (years)	16.40 ± 9.50 (8–41)	21.69 ± 12.43 (7–47)	16.55 ± 9.58 (8–47)	18.93 ± 7.71 (9–35)
Sex (male/female)	28/11	14/12	23/19	8/6
Medical history				
Age at onset of symptoms (years)	14.93 ± 9.30 (6–39)	20.42 ± 12.24 (6–43)	15.36 ± 9.53 (4–46)	/
Course of disease				
<1 year (no. of patients/eyes)	19/34	9/10	17/33	/
≥1 year (no. of patients/eyes)	20/36	17/22	25/48	/
Dry eye examination				
TMH	0.189 ± 0.052	0.170 ± 0.020	0.167 ± 0.062	/
TBUT	5.305 ± 2.632	4.770 ± 2.057	5.478 ± 2.588	/
Bular redness nasal	1.64 ± 0.76	1.52 ± 0.62	1.30 ± 0.70	/
Bulbar redness temporal	1.71 ± 0.74	1.52 ± 0.60	1.38 ± 0.64	/

NC, normal controls; TBUT, tear film breakup time; TMH, tear meniscus height.

Values are mean ± standard deviation (range) or number.

### Factors Influencing the POV Score

Ordinal logistic regression analysis using GEEs was performed to evaluate the factors influencing the visibility score of POV. The results (Table 2), indicated that age, disease groups, disease duration, and POV quadrant significantly affected POV scores. Increasing age was associated with lower POV scores (*P* = 0.038). Compared with normal controls, patients in the disease group (VKC, BKC, and AC; all *P* < 0.001) had lower POV scores. A longer disease duration (≥1 year) was also associated with reduced POV visibility than those with a duration of less than 1 year. In contrast, POV morphology was more pronounced in the superior and inferior quadrants than in the nasal and temporal quadrants (*P* < 0.001). No significant associations were observed between POV visibility and laterality (left or right eye; *P* = 0.268) or sex (*P* = 0.155). Notably, the inclusion of dry eye–related parameters (tear film breakup time, tear meniscus height, and conjunctival redness) in the model did not significantly influence the POV scores (all *P* > 0.05).

**Table 2.** Factors That Positively or Negatively Affected the POV Grade Upon Ordinal Logistic Regression Analysis

				95% Confidence Interval	
	Reference Standard	*P* Value	Odds Ratio	Lower Limit	Upper Limit
Disease					
VKC	NC	<0.001	0.098	0.053	0.182
BKC	NC	<0.001	0.095	0.052	0.177
AC	NC	<0.001	0.231	0.131	0.406
Duration					
<1 year	≥1 year	<0.001	5.093	3.077	8.431
Position					
Superior	Nasal/temporal	<0.001	10.676	6.851	16.636
Inferior	Nasal/temporal	<0.001	23.527	15.874	34.870

**Figure 2.**
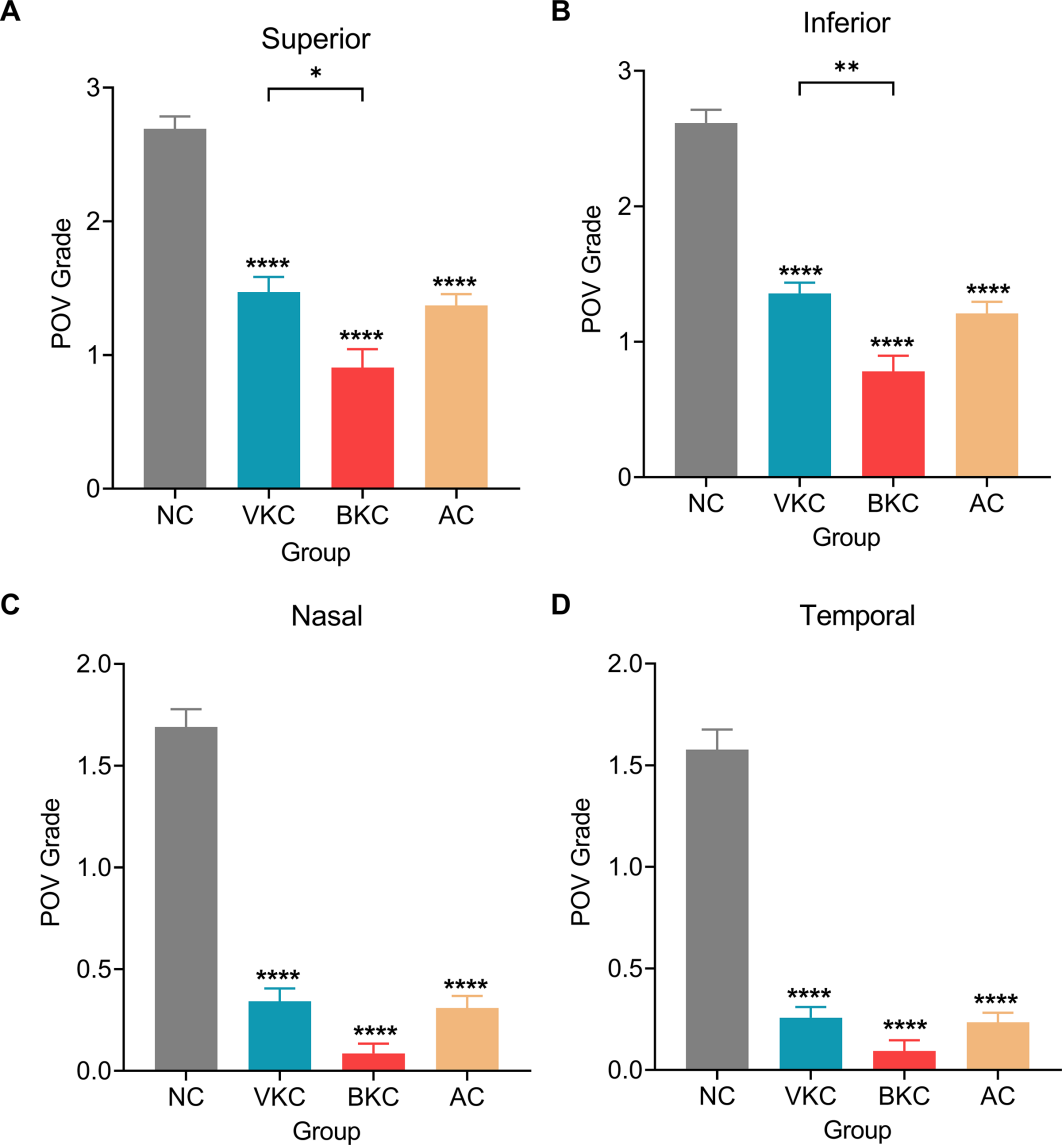
The POV visualization degree between VKC, BKC, and AC groups and normal controls (NCs). The graph revealed that the POV visualization degree in disease groups (AC, VKC, BKC) was significantly lower than the NCs in the superior (**A**), inferior (**B**), nasal (**C**), and temporal (**D**) quadrants. *P < 0.05; **P < 0.01; ****P < 0.0001; compared with the control group. Statistical analysis was performed using the Kruskal–Wallis one-way analysis of variance test.

**Figure 3.**
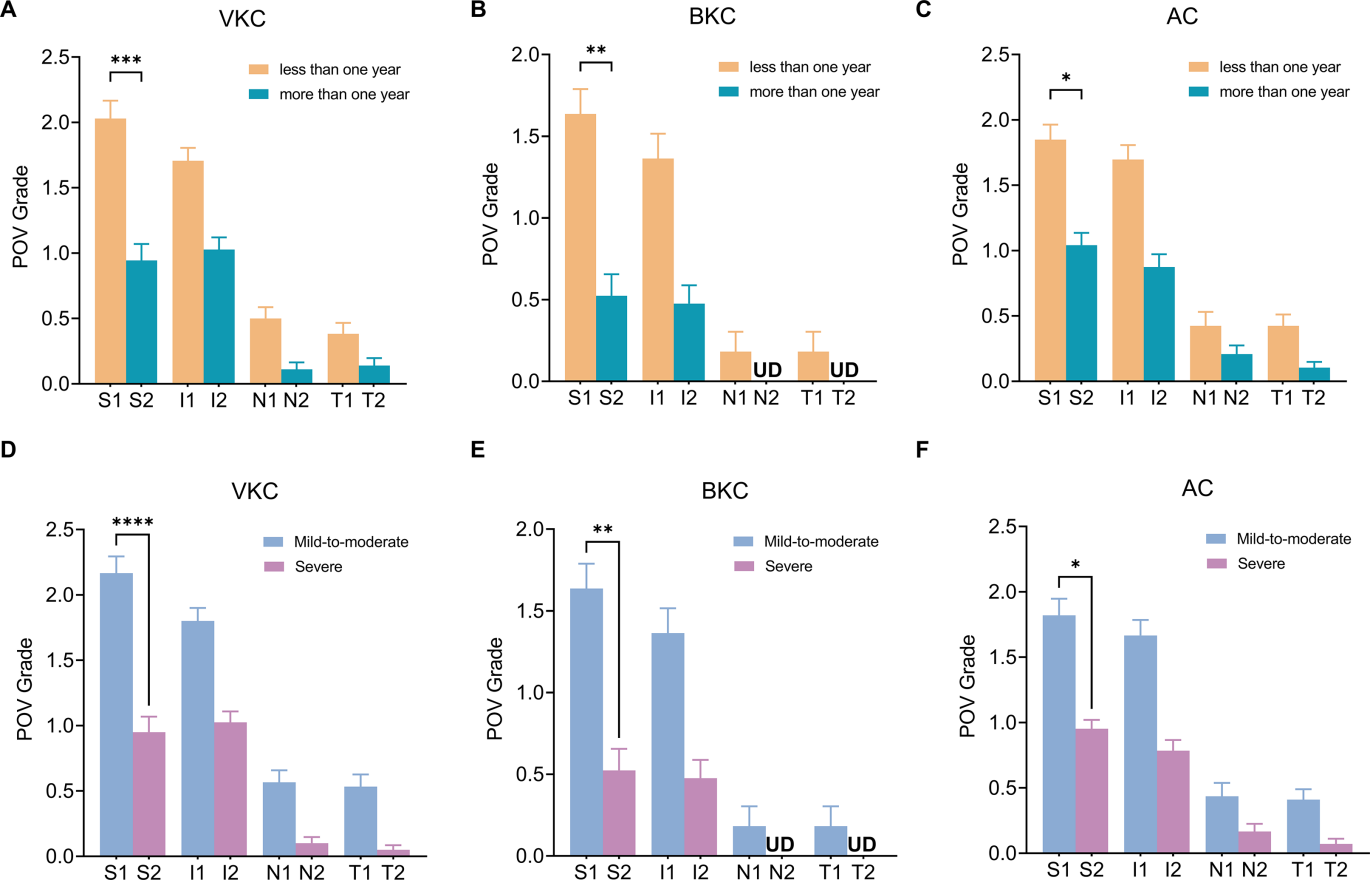
Comparison of POV visualization degree stratified by disease duration and severity in VKC, BKC, and AC. The POV visualization degree was compared according to disease duration (<1 year vs ≥1 year) and disease severity (mild-to-moderate vs severe) in VKC (**A**, **D**), BKC (**B**, **E**), and AC (**C**, **F**). Across all three disease types, lower POV visualization tended to be observed in patients with medical history lasted more than 1 year and in those with more severe disease. This pattern was consistent across different limbal regions, with more pronounced differences in the superior quadrant. *P < 0.05; **P < 0.01; ***P < 0.001. UD, undetected. Statistical analysis was performed using the Kruskal–Wallis one-way analysis of variance test.

To further characterize these associations, subgroup and stratified analyses were performed. Compared with normal controls, VKC, BKC, and AC patients showed significantly reduced POV visibility across all quadrants (Fig. 2), with more pronounced morphology in the superior and inferior quadrants. Among the disease groups, BKC exhibited lower POV scores than VKC in the superior and inferior quadrants, and no significant difference was observed between VKC and AC. Age-stratified analyses (Table 3) showed consistent findings, with a similar quadrant pattern and an overall decrease in POV visibility with increasing age. BKC also showed lower POV scores across age groups. In addition, lower POV scores were observed in patients with a disease duration of 1 year or more and in those with more severe disease (Fig. 3), particularly in the superior quadrant.

**Table 3.** Variances of POV Grade in Patients and NCs in the Different Age Groups

			POV					
Group	Age, Years	No	Superior	Inferior	Nasal	Temporal	H	*P* Value
VKC	≤10	25	1.32 ± 1.06	1.48 ± 0.42	0.20 ± 0.17	0.24 ± 0.19	49.488	<0.001
	11–20	25	1.36 ± 0.82	1.32 ± 0.39	0.32 ± 0.23	0.16 ± 0.14	47.126	<0.001
	>20	20	1.80 ± 0.70	1.25 ± 0.51	0.40 ± 0.25	0.40 ± 0.25	35.711	<0.001
BKC	≤10	10	1.20 ± 0.62	0.80 ± 0.62	0.20 ± 0.18	0.20 ± 0.18	12.65	<0.01
	11–20	5	1.00 ± 1.00	0.80 ± 0.20	0.00 ± 0.00	0.00 ± 0.00	9.868	<0.05
	>20	17	0.71 ± 0.47	0.76 ± 0.44	0.00 ± 0.00	0.00 ± 0.00	29.43	<0.001
AC	≤10	27	1.67 ± 0.69	1.44 ± 0.87	0.41 ± 0.48	0.41 ± 0.25	42.715	<0.001
	11–20	31	1.39 ± 0.38	1.29 ± 0.35	0.39 ± 0.25	0.16 ± 0.14	67.206	<0.001
	>20	23	1.00 ± 0.55	0.83 ± 0.42	0.04 ± 0.04	0.13 ± 0.12	40.551	<0.001
NC	≤10	6	3.00 ± 0.00	3.00 ± 0.00	2.00 ± 0.00	1.67 ± 0.27	21.805	<0.001
	11–20	7	2.71 ± 0.24	2.57 ± 0.29	1.29 ± 0.24	1.43 ± 0.29	17.571	<0.001
	>20	13	2.54 ± 0.27	2.46 ± 0.27	1.69 ± 0.23	1.62 ± 0.26	22.193	<0.001

NC, normal controls.

Data are mean ± standard deviation. Statistical analysis was performed using the Kruskal–Wallis one-way analysis of variance test.

### Quantitation of Cellular Changes in the Cornea

**Figure 4.**
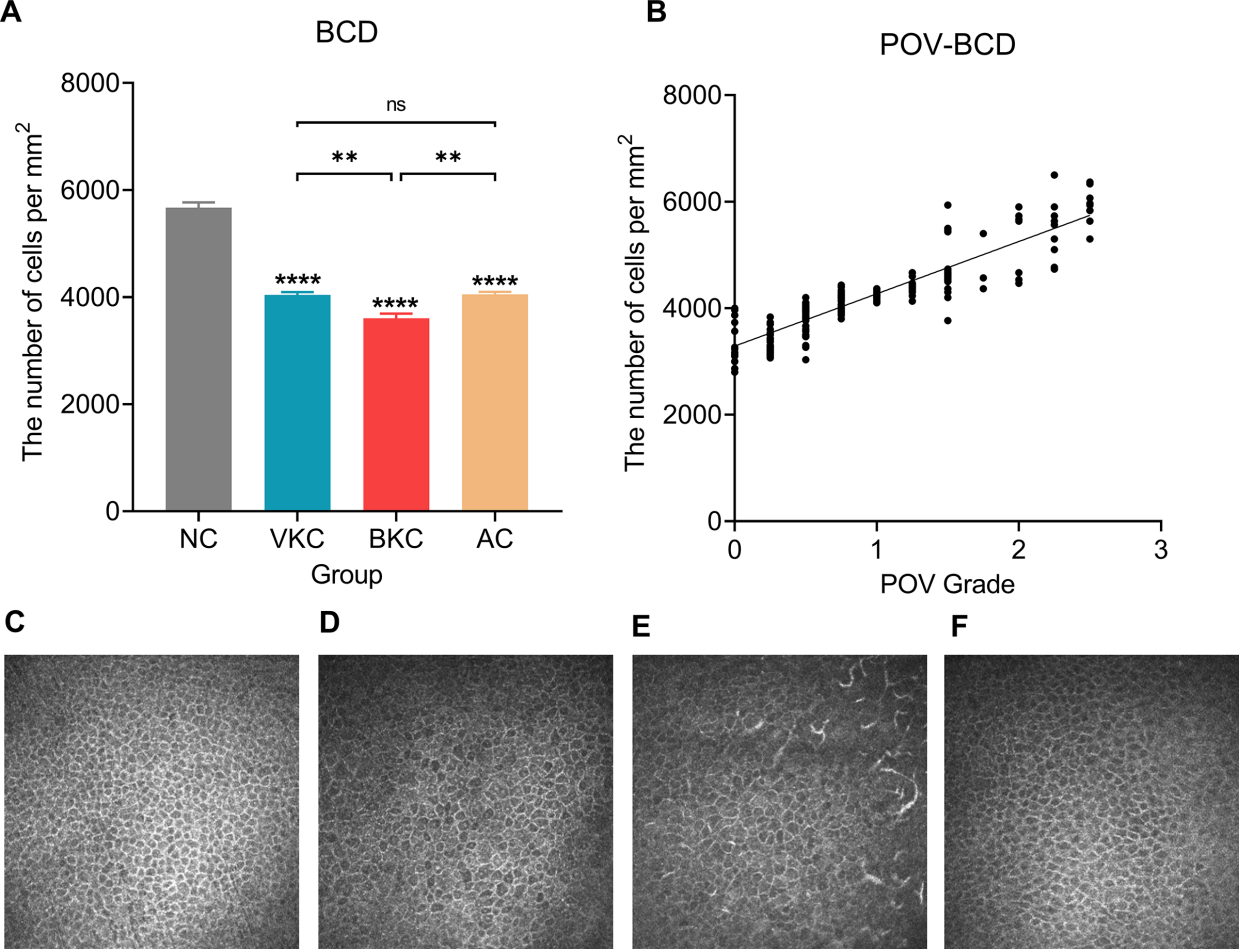
The BECD alterations in VKC, BKC, and AC groups, and normal controls (NCs). (**A**) The graph revealed that the BECD decreased in VKC, BKC, and AC groups compared with NCs. (**B**) Simple linear regression between POV and BECD parameters. (**C**–**E**) The representative confocal images of basal epithelial cells in NCs (**C**), VKC (**D**), BKC (**E**), and AC (**F**) groups. **P < 0.01; ****P < 0.0001; compared with the control group. Statistical analysis was performed using the Kruskal–Wallis one-way analysis of variance test.

To quantify the microstructural changes in the corneas associated with ocular surface inflammatory diseases, we further assessed the BECD in VKC, BKC, AC, and normal controls (Fig. 4). Compared with the normal controls, those with VKC, BKC, and AC had an average of 28.7%, 36.4% and 28.5% reduction in BECD, respectively. Moreover, we confirm the strong correlation between BECD and POV scores (*r* = 0.8033; *P* < 0.0001).

### Quantitation of SBNP Parameters

**Figure 5.**
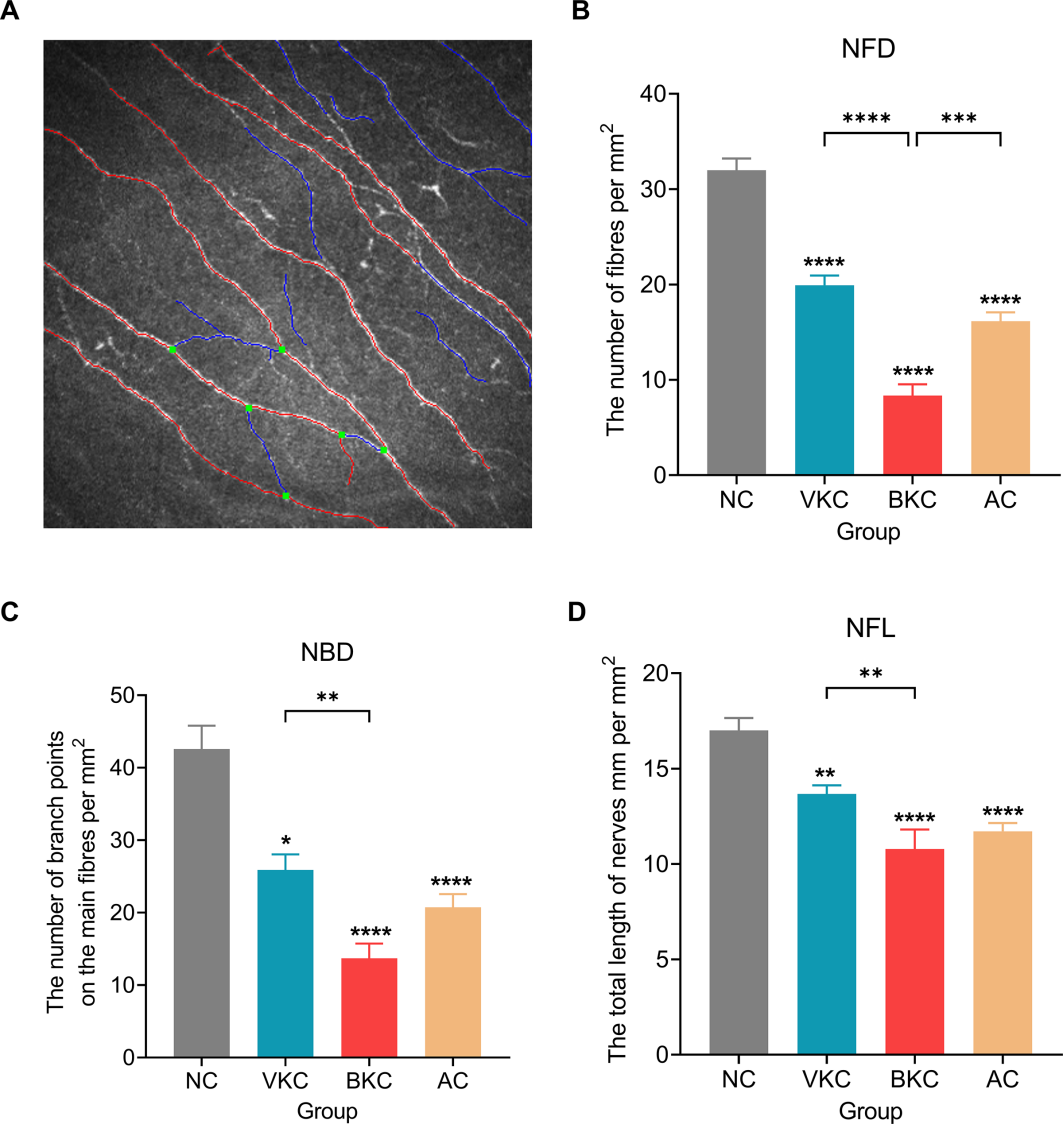
The SBNP parameters (NFD, NBD, NFL) between VKC, BKC, and AC groups and NCs. (**A**) The model diagram of neural parameters analyzed by ACCMetrics. *Red* indicates fiber, *blue* indicates branch, and *green* indicates branch point. The results showed that compared with NC, SBNP parameters in the VKC, BKC, and AC groups were significantly decreased in the aspect of the NFD (**B**), NBD (**C**), and NFL (**D**). *P < 0.05; **P < 0.01; ***P < 0.001; ****P < 0.0001. *P* values are compared with the control group. Statistical analysis was performed using the Kruskal–Wallis one-way analysis of variance test.

Differences in SBNP between the VKC, BKC, and AC groups and normal controls were analyzed in terms of the NFD, NBD, and NFL index (Fig. 5). Compared with normal controls, all three disease groups exhibited significant reductions in SBNP parameters (*P* < 0.05), including decreased NFD, NBD, and NFL. Among the disease groups, the BKC group showed the most pronounced reductions in SBNP parameters compared with the VKC and AC groups.

Spearman correlation analysis was used to analyze the correlation between POV and SBNP neural parameters (Table 4), and POV grading was calculated using the scores in the upper, lower, nasal, and temporal quadrants of each eye. The results showed that POV had a strong correlation with SBNP neural parameters NFD, NBD, and NFL (*P* < 0.0001).

**Table 4.** Spearman Correlation Analysis Between POV and SBNP Neural Parameters

			95% Confidence Interval	
POV	*r*	*P* Value	Lower Limit	Upper Limit
NFD	0.807	<0.001	0.752	0.851
NBD	0.761	<0.001	0.696	0.814
NFL	0.733	<0.001	0.661	0.791

### Corneal Microstructure Variance in VKC, BKC, and AC Patients

**Figure 6.**
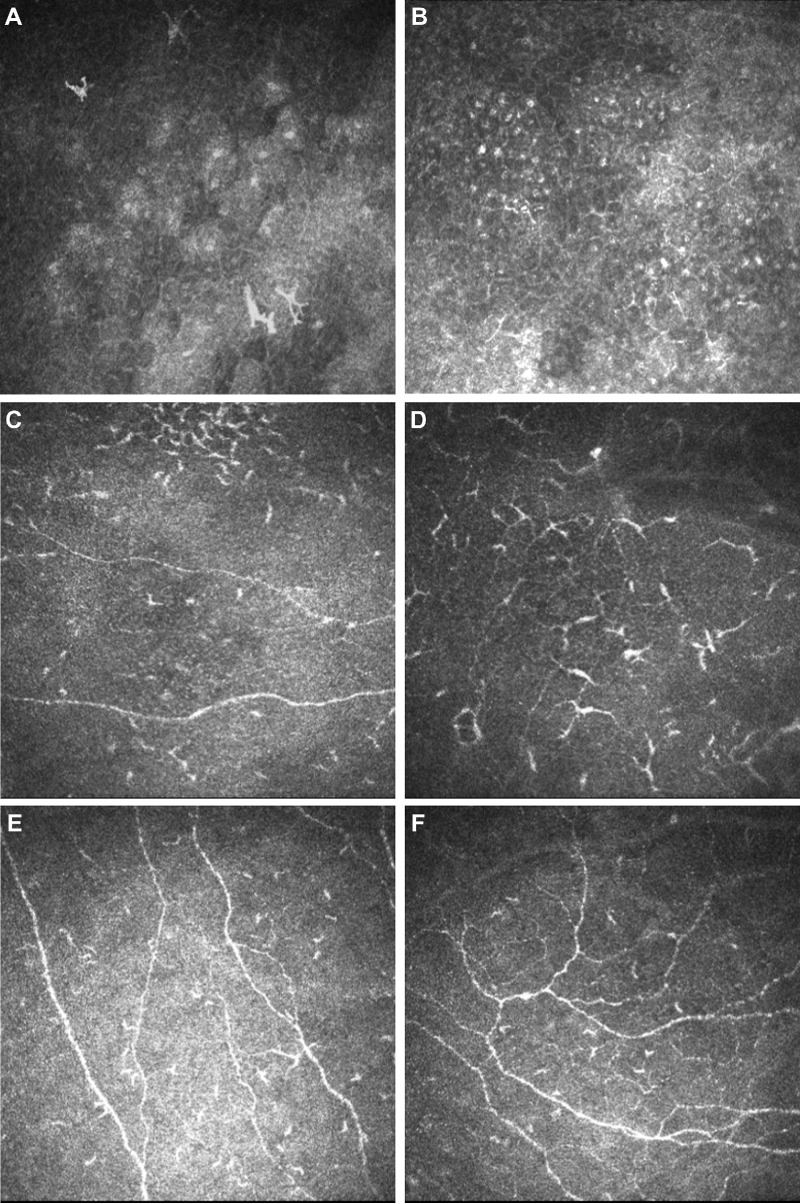
The representative confocal images of corneal microstructure in VKC and AC patients. (**A**–**D**) VKC patients, indicating a large cell diameter with high reflectivity in the (**A**) superficial and (**B**) basal epithelium, decreased subepithelial nerve density and increased activated dendritic cells (**C**, **D**), (**E**, **F**) AC patients, tortuous subcutaneous nerve and activated dendritic cells were seen (**E**, **F**).

The findings of this study indicated a significant involvement of corneal epithelial cells and SBNP in VKC to a large extent. VKC patients exhibited notable characteristics, such as enlarged cell diameter, enhanced reflectance, and nuclear activation in both the superficial epithelial cell layer and basal cell layer. In addition, a decrease in sub-basal nerve density, an increase in contortions, and an increase in activated dendritic cells were discovered in both VKC and AC patients (as present in Fig. 6).

**Figure 7.**
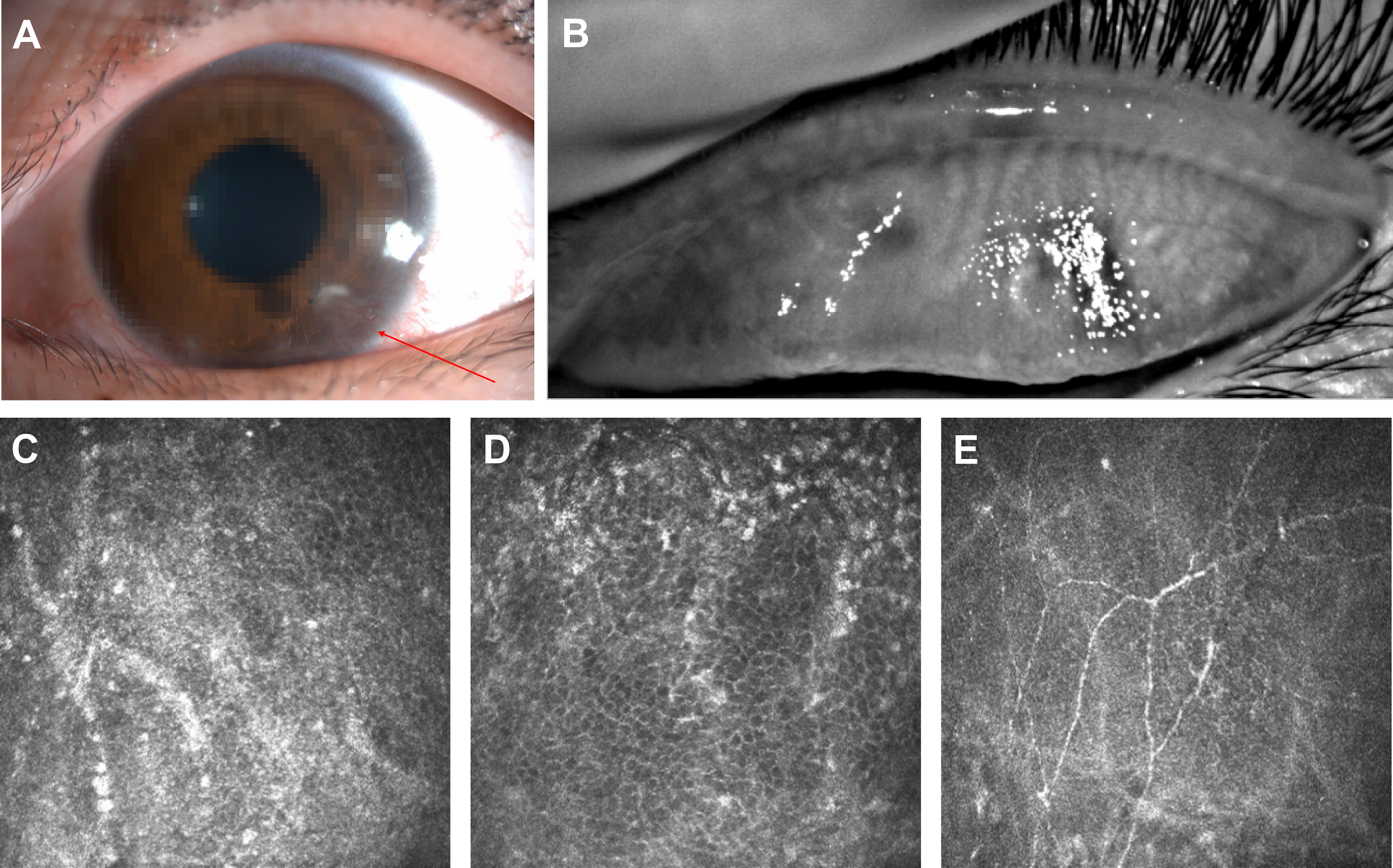
Representative slit-lamp image and infrared imaging of the meibomian gland and confocal images of corneal microstructure in an 8-year-old BKC patient. The slit-lamp image indicated corneal inflammation (**A**) in the BKC patient, marked by a *red arrow*. Infrared imaging of the meibomian gland revealed typical obstruction of the meibomian gland opening as well as atrophy and absence of meibomian glands (**B**) in the BKC patient. Worse visibility of POV (**C**, **D**) and decreased corneal sub-basal nerve density (**E**) were also seen.

The research findings indicated a more unfavorable state of POV visibility and reduced corneal sub-basal nerve density among patients diagnosed with BKC. Furthermore, a majority of these patients had experienced meibomian gland atrophy and loss (as present in Fig. 7).

### Alterations of POV Morphology After Treatment With Tacrolimus Eye Drops in VKC Patients

**Figure 8.**
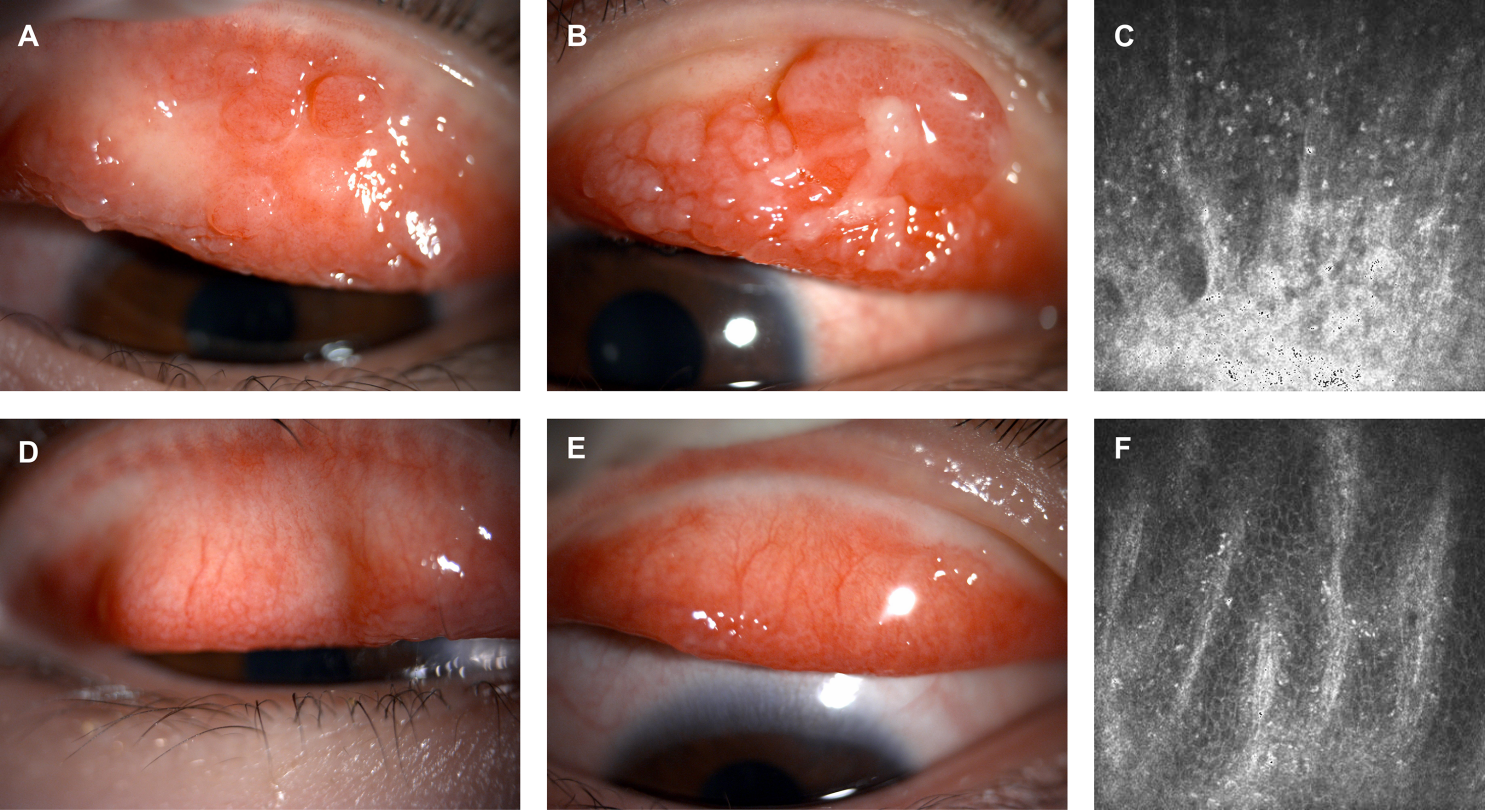
The representative slit-lamp and confocal images in VKC patients before and after 4 months of treatment with tacrolimus. (**A**–**C**) Untreated VKC patients, indicating giant gelatinous papilla lesions of palpebral conjunctival (A and B), as well as the fuzzy limbal paradise of Vogt (**C**). (**D**–**F**) VKC patients after 4 months of treatment with tacrolimus eye drops. The papillary lesions of palpebral conjunctival visibly subsided (**D** and **E**), with the morphology of POV slightly improved (**F**).

After 4 months of treatment with tacrolimus eye drops in VKC patients, clinical signs such as eye irritation and gelatinous papillary lesions of the palpebral conjunctiva and corneal limbus showed some improvement, as shown in Figure 8. A trend toward a slight improvement in POV morphology was also observed compared with untreated patients (Table 5), although these changes did not attain statistical significance (*P* > 0.05).

**Table 5.** Alterations of POV Score and Dry Eye Index After Treatment With Tacrolimus Eye Drops for 4 Months

		POV					
Group	Treated	Superior	Inferior	Nasal	Temporal	TMH	BUT
VKC	Before	1.62 ± 1.00	1.39 ± 0.79	0.42 ± 0.49	0.35 ± 0.48	0.18 ± 0.05	5.84 ± 2.25
	After	1.89 ± 1.05	1.69 ± 0.67	0.58 ± 0.63	0.46 ± 0.63	0.18 ± 0.04	6.15 ± 3.33

Values are mean ± standard deviation.

## Discussion

In this study, we used IVCM to observe relevant ocular surface indicators such as the presence of POV, BECD, and SBNP in VKC, BKC, and AC patients. Our findings demonstrated that chronic ocular surface inflammatory diseases are associated with significant structural and microenvironmental changes in the LSC niche, characterized by reduced POV visibility, decreased BECD, and impaired corneal innervation. These alterations were further influenced by age, disease duration, and limbal location, highlighting the multifactorial nature of limbal niche morphology.

Imaging and quantitative assessment of POV, BECD, and SBNP using IVCM have been reported in several studies, particularly in patients afflicted with LSCD. Le et al.^28^ demonstrated that the POV structure is markedly deteriorated in LSCD and that the presence of the POV is significantly correlated with limbal epithelial thickness. Additionally, Deng et al.^32^ found that the reduction in corneal BECD in LSCD is significantly associated with a decrease in SBNP density. These findings suggest that POV, BECD, and SBNP are three key indicators for evaluating the severity of LSCD, and they collectively reflect limbal epithelial health. To our knowledge, this study is the first to use IVCM to quantitatively evaluate POV, BECD, and SBNP parameters in patients with VKC, BKC, and AC to investigate changes in corneal structure in these related diseases and analyze the factors contributing to POV morphology.

In our study, reduced POV visibility was consistently observed across all disease groups, suggesting that chronic inflammation may disrupt the integrity of the LSC niche. Notably, advancing age was associated with lower POV scores. This finding is consistent with previous reports by Notara et al.,^33^ which demonstrated age-related flattening of limbal crypt structures and reduced proliferative capacity of limbal epithelial cells. This finding supports the concept that aging and inflammation may synergistically contribute to the deterioration of the limbal niche.

In addition, we observed a distinct spatial pattern, with more prominent POV morphology in the superior and inferior quadrants compared with the nasal and temporal regions. This regional variation is in line with previous studies by Le et al.^28^ and may reflect inherent differences in limbal epithelial thickness and stem cell distribution. Such spatial heterogeneity of the limbus may reflect inherent variations in LSC function and microenvironmental regulation. In this context, pterygium has been increasingly recognized as a complex ocular surface disorder involving aberrant proliferation and centripetal extension of limbal epithelial cells, accompanied by disruption of Bowman's layer, epithelial–mesenchymal transition, stromal fibrovascular activation, inflammation, and extracellular matrix remodeling.^34^ These pathological processes are driven by a network of cytokines, growth factors, and matrix metalloproteinases and are closely associated with localized LSC dysfunction. The nasal predominance of pterygium has been partly explained by peripheral light focusing effects of the cornea, which may increase localized ultraviolet exposure and contribute to regional vulnerability of the limbal niche.^35^

This study also reported decreased POV scores in patients with VKC, BKC, and AC, particularly in the superior limbal quadrant, as well as in those with a longer disease duration and greater disease severity. These findings suggest that a longer disease duration and greater severity are associated with more pronounced damage to POV structures. Furthermore, our results revealed that patients who suffer from VKC, AC, and BKC experience injuries to the POV structure in all quadrants, along with decreased BECD. These changes may reflect corneal stem cell dysfunction associated with chronic ocular surface inflammation.^36^ Clinically, this work highlights the potential importance of early intervention to preserve POV morphology and LSC function. Although disease severity was assessed in this study, its close relationship with disease duration makes it difficult to fully disentangle their individual effects. Additionally, although dry eye-related factors in the models did not significantly associate with POV scores, residual confounding from unmeasured or subtle ocular surface factors cannot be entirely excluded. Future studies using comprehensive assessment of ocular surface status are warranted to further clarify these relationships.

From a mechanistic perspective, it is commonly accepted that the equilibrium of the corneal epithelium is maintained by LSCs.^37^ When LSCs are partially or completely damaged, the conjunctival epithelium can migrate to the corneal surface, leading to a pathological state known as corneal conjunctivization.^38^ The process of conjunctivization is associated with destruction of the basement membrane of the limbal cornea and a decline in the POV structural score.^39^ However, it is worth mentioning that the absence of the POV or the presence of corneal neovascularity does not necessarily indicate the absence of LESCs. Even in cases without a typical POV structure, residual normal limbal epithelial cells have been observed in eyes with full LSCD clinical features.^40^ These cells are characterized by their small cell size, dark cytoplasm, and distinct cell–cell borders arranged in a regular pattern.^41^

In addition to alterations in POV morphology, our study demonstrated significant reductions in BECD and SBNP parameters across all disease groups. Previous studies have shown a negative correlation between BECD and epithelial thickness with the clinical stage of LSCD.^12^^,^^42^ Consistently, we observed a concordant decrease in central BECD and POV scores in patients compared with normal controls. This reduction in BECD was accompanied by a widespread increase in basal cell size, suggesting a loss of the LSC population and limited capacity to compensate for cellular damage through proliferation, which may ultimately lead to depletion of the remaining stem cell pool and progression toward LSCD. Therefore, the limbal crypt structure together with BECD help us to better understand the LSC alterations and hold guiding implications in assessing the quantity and functionality of LSCs.

We further analyzed SBNP parameters, including NFD, NBD, and NFL, and found that all were significantly reduced in the disease groups compared with controls. Corneal nerves play a critical role in maintaining epithelial health by providing trophic support and promoting wound healing. The observed reduction in SBNP parameters aligns with previous findings showing a negative correlation between the density of the corneal SBNP and conjunctivization of the LSCD.^43^ Moreover, we found a strong and statistically significant correlation between the POV and the NFD, NBD, and NFL. This result suggests that, although they represent two different pathophysiological processes, POV and SBNP have a high level of correlation, which collectively offers valuable insights for the diagnosis of LSCD.

BKC is an inflammatory condition affecting the surface of the eyes and eyelids, which involves changes in the eyelids, dysfunction of the meibomian glands, and inflammation of the conjunctiva and cornea. Prolonged inflammation of the cornea can result in scarring, vascularization, and opacity. It is crucial to correctly diagnose BKC in clinical practice to prevent severe visual impairment. Recent studies have highlighted the close association between BKC and a history of meibomian gland dysfunction.^44^ Compared with patients with meibomian gland dysfunction–induced hyperevaporative dry eye, individuals with BKC experience more severe impairment in both the function and structure of the meibomian gland, as evidenced by higher meibomian gland dropouts and poorer meibum quality.^44^ This study also revealed significant loss of the meibomian gland in most BKC patients with meibomian gland dysfunction, accompanied by worse limbal niche and decreased corneal sub-basal nerve density. These findings suggest that the keratoconjunctival damage associated with blepharitis may lead to ocular surface microstructural alterations, underscoring the importance of prompt treatment.

Previous studies using IVCM have demonstrated that VKC is associated with widespread corneal microstructural alterations, affecting nearly all corneal layers except the endothelium.^45^ Reported changes include epithelial cell activation, reduced corneal cell density, increased inflammatory cell infiltration, and alterations in SBNP parameters, such as reduced nerve density and increased tortuosity.^45^ In AC, increased density and activation of dendritic cells have also been observed, reflecting an upregulated ocular surface immune response.^46^ Notably, some of these microstructural changes have been shown to be partially reversible after anti-inflammatory treatment, such as topical cyclosporine, which has been associated with reduced epithelial activation and inflammatory cell density, as well as modifications in nerve fiber parameters.^47^ Consistent with these findings, our results further support the presence of inflammation-associated microstructural changes in allergic ocular surface diseases, particularly involving sub-basal nerve alterations and increased immune cell activation. These changes may contribute to the disruption of the corneal microenvironment and potentially affect the LSC niche, which is critical for maintaining epithelial homeostasis.

From a clinical perspective, VKC is characterized by distinct features such as Horner–Trantas spots, which appear as yellow–white deposits composed of degenerated eosinophils and epithelial cells as well as giant papillae (>1 mm) on the upper tarsal conjunctiva and gelatinous limbal papillae.^3^ These findings reflect intense and chronic eosinophil-driven inflammation at the ocular surface, particularly involving the limbal region. Previous studies have suggested that the presence of large papillae and gelatinous limbal involvement may be associated with poorer clinical outcomes, highlighting the link between sustained inflammation and disease severity.^48^ In addition, findings such as pseudogerontoxon and perilimbal hyperpigmentation further indicate long-standing alterations in limbal vascular permeability and tissue remodeling.^3^^,^^49^^,^^50^ Collectively, these clinical manifestations underscore the persistent inflammatory burden at the limbus, which may contribute to the structural and functional disruption of the LSC niche. Such alterations are consistent with the microstructural changes observed in our study, including changes in POV morphology, supporting the role of POV as a marker of limbal niche integrity.

The AC group in this study comprised both seasonal AC and perennial AC, which may introduce heterogeneity in disease characteristics and inflammatory severity. VKC was analyzed separately because of its greater clinical severity and more pronounced corneal involvement. Although this grouping may limit the ability to detect subtype-specific differences, it is unlikely to substantially influence the overall comparison between the VKC and non-VKC groups. Moreover, further studies with larger cohorts are warranted to enable more detailed subtype-specific analyses.

This study also explored changes in clinical signs and POV morphology in VKC patients treated with tacrolimus eye drops. However, the observed changes in POV morphology after tacrolimus therapy were exploratory and did not attain statistical significance. These findings highlight the challenges in reversing structural changes in the POV over a short treatment period. The small sample size, short follow-up duration, and pilot nature of this analysis limit the conclusions that can be drawn regarding the long-term effects of tacrolimus on the LSC niche. Future studies with larger cohorts and extended follow-up are needed to determine whether tacrolimus can meaningfully influence POV morphology and potentially mitigate progression toward LSCD.

This study also presents several limitations. First, POV grading was based on a semiquantitative, observer-dependent scale. Although grading was performed in a masked manner, reproducibility was not formally assessed, which may introduce subjective bias. Nevertheless, this approach is consistent with prior IVCM-based studies using similar observer-dependent grading strategies.^28^ Future studies with repeated or independent gradings are warranted to confirm robustness. Second, previous research has demonstrated that the SBNP exhibits a distinctive spiral or eddy pattern radiating toward the center of the cornea.^51^ However, the field of view in a single IVCM image is restricted, making it challenging to analyze the overall distribution of corneal nerves. Although we have made efforts to select nonoverlapping images with the widest nerve distribution for SBNP analysis, this approach still falls short of providing a comprehensive view. A recent study using a standardized region of interest and generating wide-area SBNP mosaics in patients with type 2 diabetes found that the NFL was 34% lower in region of interest–based analysis compared with wide-area analysis.^52^ This discrepancy suggests a notable bias and it should be noted that the pattern of nerve distribution outside the central cornea varied across different quadrants.^52^ Nonetheless, the acquisition of a wide-area SBNP image remains labor intensive and time consuming, which limits its widespread clinical application. Last, the sample size in this study is still limited, and conducting research with a more expansive sample size may yield a greater abundance of clinical data.

In summary, these findings highlight the potential of IVCM as a noninvasive tool for the early detection of limbal niche impairment in inflammatory ocular surface diseases. Our study demonstrates that severe ocular surface inflammation is associated with changes in both the LSC niche and the corneal sub-basal nerves. Moreover, age, disease duration, and limbal quadrant location appear to influence the morphological evaluation of POV. Collectively, these results enhance our understanding of the pathological alterations underlying VKC, BKC, and AC and may inform strategies for early detection of LSCD associated with these conditions.
